# Respiratory proteins contribute differentially to *Campylobacter jejuni*’s survival and in vitro interaction with hosts’ intestinal cells

**DOI:** 10.1186/1471-2180-12-258

**Published:** 2012-11-13

**Authors:** Issmat I Kassem, Mahesh Khatri, Malak A Esseili, Yasser M Sanad, Yehia M Saif, Jonathan W Olson, Gireesh Rajashekara

**Affiliations:** 1 Department of Veterinary Preventive Medicine, Food Animal Health Research Program, Ohio Agricultural Research and Development Center, The Ohio State University, Wooster, OH, 44691, USA; 2 Department of Microbiology, North Carolina State University, Raleigh, NC, 27695, USA

**Keywords:** *Campylobacter jejuni*, Respiratory proteins, Survival, Adaptation, Motility, Oxidative stress, Biofilm, Oxygen, Temperature, INT-407, Chicken intestinal epithelial cells

## Abstract

**Background:**

The genetic features that facilitate *Campylobacter jejuni*’s adaptation to a wide range of environments are not completely defined. However, whole genome expression studies showed that respiratory proteins (RPs) were differentially expressed under varying conditions and stresses, suggesting further unidentified roles for RPs in *C*. *jejuni*’s adaptation. Therefore, our objectives were to characterize the contributions of selected RPs to *C*. *jejuni*’s i- key survival phenotypes under different temperature (37°C vs. 42°C) and oxygen (microaerobic, ambient, and oxygen-limited/anaerobic) conditions and ii- its interactions with intestinal epithelial cells from disparate hosts (human vs. chickens).

**Results:**

*C*. *jejuni* mutant strains with individual deletions that targeted five RPs; nitrate reductase (Δ*napA*), nitrite reductase (Δ*nrfA*), formate dehydrogenase (Δ*fdhA*), hydrogenase (Δ*hydB*), and methylmenaquinol:fumarate reductase (Δ*mfrA*) were used in this study. We show that only the Δ*fdhA* exhibited a decrease in motility; however, incubation at 42°C significantly reduced the deficiency in the Δ*fdhA*’s motility as compared to 37°C. Under all tested conditions, the Δ*mfrA* showed a decreased susceptibility to hydrogen peroxide (H_2_O_2_), while the Δ*napA* and the Δ*fdhA* showed significantly increased susceptibility to the oxidant as compared to the wildtype. Further, the susceptibility of the Δ*napA* to H_2_O_2_ was significantly more pronounced at 37°C. The biofilm formation capability of individual RP mutants varied as compared to the wildtype. However, the impact of the deletion of certain RPs affected biofilm formation in a manner that was dependent on temperature and/or oxygen concentration. For example, the Δ*mfrA* displayed significantly deficient and increased biofilm formation under microaerobic conditions at 37°C and 42°C, respectively. However, under anaerobic conditions, the Δ*mfrA* was only significantly impaired in biofilm formation at 42°C. Additionally, the RPs mutants showed differential ability for infecting and surviving in human intestinal cell lines (INT-407) and primary chicken intestinal epithelial cells, respectively. Notably, the Δ*fdhA* and the Δ*hydB* were deficient in interacting with both cell types, while the Δ*mfrA* displayed impairments only in adherence to and invasion of INT-407. Scanning electron microscopy showed that the Δ*hydB* and the Δ*fdhA* exhibited filamentous and bulging (almost spherical) cell shapes, respectively, which might be indicative of defects in cell division.

**Conclusions:**

We conclude that the RPs contribute to *C*. *jejuni*’s motility, H_2_O_2_ resistance, biofilm formation, and in vitro interactions with hosts’ intestinal cells. Further, the impact of certain RPs varied in response to incubation temperature and/or oxygen concentration. Therefore, RPs may facilitate the prevalence of *C*. *jejuni* in a variety of niches, contributing to the pathogen’s remarkable potential for adaptation.

## Background

*Campylobacter jejuni*, a Gram-negative bacterium, is a leading cause of foodborne gastroenteritis [1]. In addition, *C*. *jejuni* infections are associated occasionally with serious neuropathies and other significant sequelae in humans [1]. Historically, this bacterium has been considered fastidious, requiring microaerobic atmosphere and complex media for optimal growth under laboratory conditions. However, *C*. *jejuni* has been isolated from a variety of animals, such as poultry and cattle, as well as other ex vivo niches [2,3], which highlight the remarkable capability of this bacterium for persistence in different environments as well as its adaptation potential. Despite lacking classical stress response mechanisms [4], *C*. *jejuni* has disparate traits that promote its adaptability, including a competency for natural transformation and a highly branched respiratory chain [5,6]. The latter is composed of individual respiratory proteins (RPs) that impact vital functions in *C*. *jejuni*, spanning growth and host colonization [5,7-11]. The RPs include formate dehydrogenase, hydrogenase, fumarate reductase, nitrate and nitrite reductases, and others that facilitate the transfer of electrons (from donors to acceptors), which drives respiration and, as such, energy metabolism in *C*. *jejuni*[5,11]. Further, whole genome expression studies and other transcriptional analyses showed that genes encoding RPs were differentially expressed in response to shifts in temperature, pH, and oxygen concentration [7,12-14]. Additionally, many RPs in *C*. *jejuni* are transported via the twin-arginine translocation (Tat) system [11], which is specialized in the translocation of pre-folded substrates, including cofactor containing redox proteins, across the cytoplasmic membrane. Of relevant interest is the impairment of the Tat function in *C*. *jejuni*, which leads to pleiotropic phenotypes, including defects in motility, biofilm formation, flagellation, resistance to oxidative stress, and chicken colonization [15]. These phenotypes are likely the result of multiple additive effects caused by defects in translocation of the Tat substrates, including RPs. Taken together, these observations further suggest that RPs might impact various adaptation and survival phenotypes in *C*. *jejuni*. However, beyond the aforementioned studies and the role of RPs in *C*. *jejuni*’s respiration, little is known about the contributions of these proteins to the success of *C*. *jejuni* under changing environmental conditions; a property that is critical for understanding the transmission of this pathogen between environments and hosts. Therefore, in this study, we describe the role of five RPs that were predicted to be Tat-dependent [15] in *C*. *jejuni*’s motility, resistance to hydrogen peroxide (H_2_O_2_) and biofilm formation under different temperature and/or oxygen conditions. We also assessed the contribution of RPs to the bacterium’s in vitro interactions with intestinal epithelial cells of two important hosts (humans and chickens). For these purposes, *C*. *jejuni* strains with deletion in five individual genes encoding essential RPs subunits were used. The mutations targeted the nitrate reductase (Δ*napA*; *Cj0780*), nitrite reductase (Δ*nrfA*; *Cj1357c*), formate dehydrogenase (Δ*fdhA*; *Cj1511c*), hydrogenase (Δ*hydB*; *Cj1266c*), methylmenaquinol:fumarate reductase (Δ*mfrA*; *Cj0437*; this gene was previously identified as encoding a succinate dehydrogenase subunit, *sdhA*). It was previously shown that the deletion of these genes resulted in the loss of the catalytic functions of the associated respiratory enzymes; however, the mutants retained a generation time that was similar to that of the parental strain [8-10]. Although the mutants’ role in respiration has been previously investigated, neither the impact of the cognate RPs on survival phenotypes such as H_2_O_2_ resistance and biofilm formation nor their potential contribution to adaptation under varying temperature and oxygen conditions were analyzed. Further, the potential interactions of these mutants with human and chicken intestinal cells were not characterized. Here, we show that individual RPs can contribute to *C*. *jejuni*’s motility, oxidative stress response (H_2_O_2_ resistance), biofilm formation, and in vitro interactions with host cells. Our data highlight a role for RPs in *C*. *jejuni*’s adaptation to different environmental conditions as well as its in vitro interactions with intestinal cells of disparate hosts.

## Results and discussion

**Figure 1:**
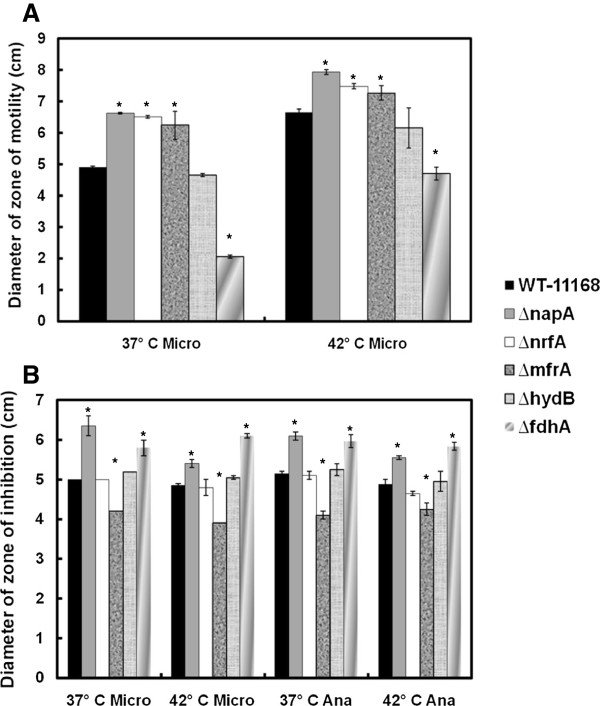
**The mutants’ impact on motility and H**_**2**_**O**_**2 **_**resistance under different incubation conditions.** (**a**) The diameter of the zone of motility was measured under different incubation temperatures and compared to the wildtype. (**b**) H_2_O_2_ resistance was assessed using a standard diffusion method. Microaerobic and anaerobic atmospheres are abbreviated as “Micro” and “Ana”, respectively. Statistically significant (*P* < 0.05) differences are highlighted with * and indicate comparisons with the wildtype. The experiment was repeated three times independently and samples were tested in triplicate per experiment. Data are presented as mean ± standard error.

*C*. *jejuni*’s motility is considered important for effective colonization of hosts as well as chemotaxis [16] and, subsequently, persistence in different niches. Therefore, we investigated whether the deletion of the RPs might differentially impact *C*. *jejuni*’s motility in response to different temperatures. Examination under scanning electron microscopy showed that none of the mutants were defective in flagellation, regardless of the incubation temperature (data not shown). Further, the mutants’ motility was evaluated using 0.4% semisolid agar as described elsewhere [15,17]. Using this method, motility under anaerobic conditions could not be accurately assessed, because the zones of motility were not defined and sufficiently large for reliable measurement. This precluded the assessment of the effect of oxygen concentration on motility. However, our results show that during incubation under microaerobic conditions, Δ*fdhA* displayed significantly decreased zone of motility as compared to the wildtype, while the deletion of *hydB* did not impact this phenotype (Figure 1a, Table 1). Alternatively, Δ*napA*, Δ*nrfA*, and Δ*mfrA* exhibited significantly increased motility as compared to the wildtype (Figure 1a, Table 1). Since the oxidation of formate is considered a major energy source for *C*. *jejuni*[18], the motility defects that are displayed by the Δ*fdhA* as compared to the other mutants and the wildtype strain can be perhaps attributed to the role of the formate dehydrogenase in energy metabolism.

**Table 1:** Summary of the phenotypes associated with the RPs mutants

**Mutant**	**Motility (Micro)**		**Res. H**_**2**_**O**_**2**_**(Micro)**		**Res. H**_**2**_**O**_**2**_**(Ana)**		**Biofilm (Micro)**		**Biofilm (Ana)**		**Biofilm (O**_**2**_**)**		**PIC (42°C)**		**INT-407 (37°C)**			**Cell shape**	
	**37°C**	**42°C**	**37°C**	**42°C**	**37°C**	**42°C**	**37°C**	**42°C**	**37°C**	**42°C**	**37°C**	**42°C**	**Adh**	**Inv**	**Adh**	**Inv**	**Intra**	**37°C**	**42°C**
**Δ*napA***	**↑**	**↑**	**↓**	**↓**	**↓**	**↓**	**NS**	**NS**	**↑**	**NS**	**↓**	**↓**	**NS**	**NS**	**↓**	**NS**	**NS**	**Normal**	
**Δ*nrfA***	**↑**	**↑**	**NS**	**NS**	**NS**	**NS**	**NS**	**NS**	**↑**	**NS**	**NS**	**NS**	**↑**	**NS**	**NS**	**NS**	**↑**	**Normal**	
**Δ*mfrA***	**↑**	**↑**	**↑**	**↑**	**↑**	**↑**	**↓**	**↑**	**NS**	**↓**	**NS**	**NS**	**NS**	**NS**	**↓**	**↓**	**NS**	**Normal**	
**Δ*hydB***	**NS**	**NS**	**NS**	**NS**	**NS**	**NS**	**NS**	**NS**	**NS**	**NS**	**NS**	**NS**	**↓**	**↓**	**↓**	**↓**	**↓**	**Filament**	
**Δ*fdhA***	**↓**	**↓**	**↓**	**↓**	**↓**	**↓**	**↓**	**NS**	**NS**	**NS**	**NS**	**NS**	**↓**	**↓**	**NS**	**↓**	**↓**	**Bulging**	

Res. H_2_O_2_ and PIC indicate resistance to hydrogen peroxide and primary chicken intestinal epithelial cells, respectively. Microaerobic, anaerobic, and ambient oxygen incubation conditions are abbreviated as “Micro”, “Ana” and “O_2_” respectively, while adherence, invasion and intracellular survival are abbreviated as “Adh”, “Inv” and “Intra”. Statistically significant increases or decreases (*P* < 0.05) as compared to the wildtype are indicated by ↑ and ↓, respectively, while NS indicates no significant differences.

Incubation at 42°C significantly increased the zone of motility for all the strains as compared to 37°C (Figure 1a, Table 1). This suggested that *C*. *jejuni*’s zone of motility was responsive to temperature, which corroborates results observed in other bacteria [19,20]. Further, although the Δ*fdhA* remained defective in motility as compared to the wildtype at 42°C, its motility zone was significantly larger at 42°C as compared to 37°C (Figure 1a, Table 1). Subsequently, our results suggest that the severity of the ramifications associated with an RP mutant’s impaired motility might be dependent on the temperature of a host or a niche (e.g. ~ 37°C human body temperature vs. the 42°C of chickens).

During its transmission between hosts and environments, *C*. *jejuni* encounters different concentrations of oxygen that range from oxygen-limited (hosts’ guts) to ambient (ex vivo) conditions, which indicates that oxidative stress resistance mechanisms are essential for the success of this pathogen. In other studies, fumarate reductase, formate dehydrogenase, and hydrogenase were found to contribute to oxidative stress responses in *Bacteroides fragilis*, *Desulfovibrio vulgaris*, and *Geobacter sulfurreducens*, respectively [21-23]. In *C*. *jejuni*, the genes encoding nitrate reductase were shown to be repressed in a PerR (peroxide stress regulator) mutant and after exposure to H_2_O_2_ and iron, which fuel the Fenton reaction and the production of hydroxyl radicals [24], while *mfrA* is oxygen regulated [7]. Consequently, we assessed the contributions of RPs to *C*. *jejuni*’s H_2_O_2_ resistance under different temperature and oxygen conditions using a standard diffusion assay [17,24]. Our results indicated that under all incubation conditions both Δ*napA* and Δ*fdhA* were significantly more sensitive to H_2_O_2,_ while Δ*mfrA* showed more resistance to the oxidant (Figure 1b) as compared to the wildtype. The altered susceptibility to H_2_O_2_ associated with different RPs, suggests that disparate RPs might be working collaboratively to maintain the homeostasis in *C*. *jejuni* during H_2_O_2_ stress. This is conceivable since in *E*. *coli* oxidized redox enzymes can lead to the formation of superoxide anions and H_2_O_2_[25].

Although the genes encoding the RPs included in this study, with the exception of *mfrA*, are known to be upregulated at 42°C [13], the higher incubation temperature did not drastically alter the observed H_2_O_2_ resistance phenotypes for four mutants (Figure 1b). However, Δ*napA*’s susceptibility was always significantly more pronounced at 37°C (Figure 1b), but the precise reasons for this temperature associated impact and its importance (e.g. in terms of human host colonization) are currently not clear.

**Figure 2:**
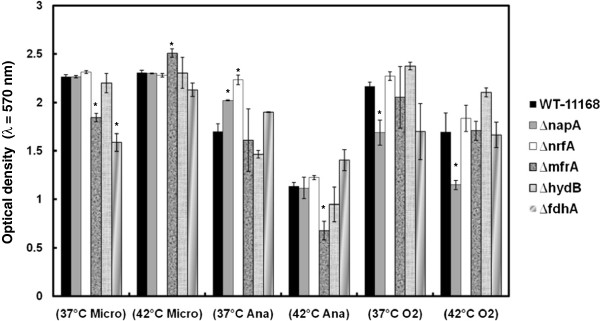
**The mutants’ impact on biofilm formation under different incubation conditions.** Microaerobic, anaerobic, and ambient oxygen incubation conditions are abbreviated as “Micro”, “Ana” and “O_2_” respectively. Statistically significant (*P* < 0.05) differences are highlighted with * and indicate comparisons with the wildtype. The experiment was repeated three times independently and samples were tested in at least three replicates per experiment. Data are presented as mean ± standard error.

Biofilm formation is an important mechanism for survival and persistence of *C*. *jejuni* in the environment [26]. Since formate dehydrogenase and nitrite reductase have been implicated in biofilm formation of two important bacterial pathogens, *Staphylococcus aureus* and *Pseudomonas aeruginosa*, respectively [27,28], we investigated the role of RPs in *C*. *jejuni*’s ability to form biofilms under different environmental conditions using the crystal violet staining assay [15,17]. Our results clearly show that RPs can impact biofilm formation in *C*. *jejuni*. For example, Δ*fdhA* and Δ*napA* were significantly deficient in biofilm formation at 37°C only in a microaerobic atmosphere and under ambient oxygen, respectively, while Δ*nrfA* and Δ*napA* displayed an increased biofilm formation at 37°C only in anaerobic conditions (Figure 2, Table 1). Therefore, our results also show that the impact of certain RPs on the biofilm phenotype was dependent on incubation temperature and/or the oxygen concentration (Figure 2, Table 1). For example, as compared to the wildtype, the Δ*mfrA* displayed significantly deficient and increased biofilm formation under microaerobic conditions at 37°C and 42°C, respectively (Figure 2, Table 1). However, under anaerobic conditions, the Δ*mfrA* was only significantly impaired in biofilm formation at 42°C (Figure 2, Table 1), while under aerobic conditions and regardless of the temperature, there were no defects in the Δ*mfrA*’s biofilms as compared to the wildtype (Figure 2, Table 1).

The observed impact of RPs on biofilm formation is likely mediated by multiple factors, including the metabolic and energy requirements that facilitate efficient growth and persistence in response to the properties of a given niche. However, our results highlight the overall importance of RPs in *C*. *jejuni*’s adaptations to different niches as well as their differential contribution to promote the pathogens survival and cognate persistence via biofilm formation in disparate environments.

**Figure 3:**
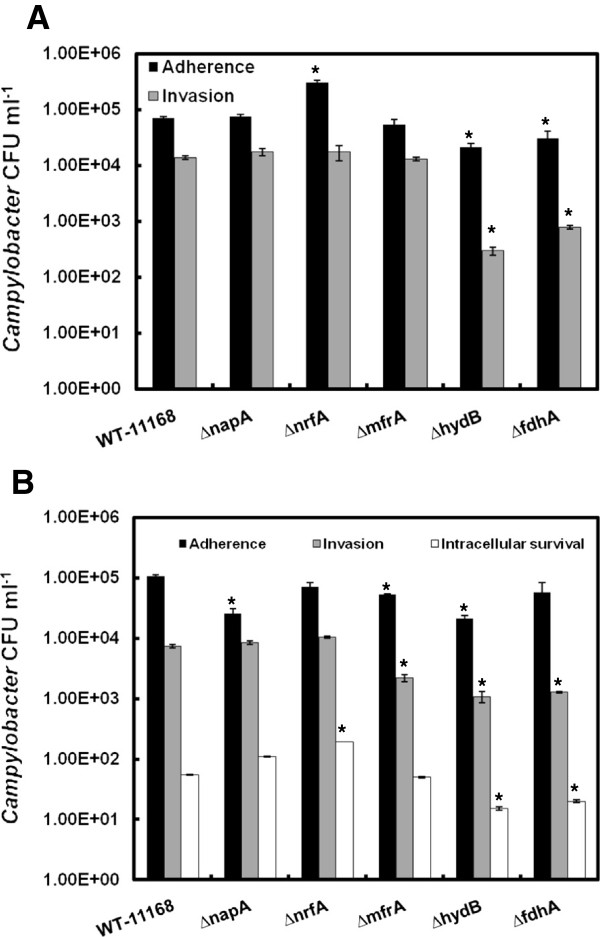
**The mutants’ interactions with PIC and INT-407 cells.** The wildtype and mutant strains were added to the monolayers to achieve a multiplicity of infection (MOI) of 1:100, respectively. (**a**) Adherence and invasion of PIC. (**b**) Adherence, invasion, and intracellular survival in INT-407. Statistically significant (*P* < 0.05) differences are highlighted with * and indicate comparisons with the wildtype. The experiment was repeated three times independently and samples were tested in duplicate per experiment. Data are presented as mean ± standard error.

Since RPs contribute to *C*. *jejuni* survival phenotypes in a manner that was dependent on the incubation temperature and/or oxygen concentration, it was important to investigate if the deletion of RPs will impact *C*. *jejuni*’s interactions with the cells of hosts that possess markedly different physiology and body temperatures. For this purpose, the interactions of the mutants with human intestinal cells (INT-407) and primary chicken intestinal epithelial cells (PIC) were analyzed using the gentamicin protection assay as described elsewhere [29,30]. All cells were incubated in a tissue culture chamber (5% CO_2_) either at 37°C or 42°C corresponding to the hosts’ body temperatures. Our results show that Δ*nrfA* adhered to PIC in significantly higher numbers, while Δ*fdhA* and Δ*hydB* were significantly deficient in adherence as well as invasion of the chicken cell monolayers (Figure 3a). While assessing intracellular survival for the mutants in PIC, no CFUs were retrieved for any of the strains, including the wildtype. This observation corroborated a previous study, which showed that during overnight incubation *C*. *jejuni* can escape the PIC monolayers due to the bacterium's inherent mode of colonization of chicken intestinal epithelia [31]. Specifically, Van Deun et al. [31] showed that *C*. *jejuni* strains that invaded PIC were not able to proliferate in the intracellular milieu and rapidly exited the cells, supposedly to replicate in the intestinal mucus. It was also suggested that this mode of infection (i.e. short-term entry to the PIC) allows *C*. *jejuni* to escape mucosal clearance [31]. In comparison to the interaction with PIC, all mutants were defective to a varying degree, albeit if not always significantly, in adherence to INT-407 cells, while Δ*mfrA*, Δ*fdhA* and Δ*hydB* were also impaired in their invasion potential and Δ*nrfA* showed an increased ability for intracellular survival (Figure 3b, Table 1). Notably, Δ*mfrA* that was not deficient in PIC showed considerable decrease in adherence to and invasion of INT-407 cells, while Δ*nrfA* did not adhere to INT-407 cells in higher numbers than the wildtype (Figure 3a and b, Table 1). Further, Δ*fdhA* and Δ*hydB* decreased potential for the invasion of the INT-407 cells was not as severe as that observed in the PIC (Figure 3a and b, Table 1). Collectively, our results suggest that under our experimental conditions the RPs contributed differentially to the virulent capabilities of *C*. *jejuni*. However, it should be noted that the use of in vitro systems in our experiment was meant only to assess the differential contribution of RPs to disparate niches and breakdown the role of these enzymes in cell adherence and invasion and intracellular survival. Therefore, extrapolations of the results to the overall outcome of in vivo colonization should be constrained. For example, it was previously shown that Δ*fdhA* and Δ*hydB* were mildly impaired in the colonization of chickens, while Δ*napA* and Δ*nrfA* were retrieved in significantly low numbers from this host [8,10]. Further, the Δ*mfrA* was not deficient in the colonization of chickens [9].

**Figure 4:**
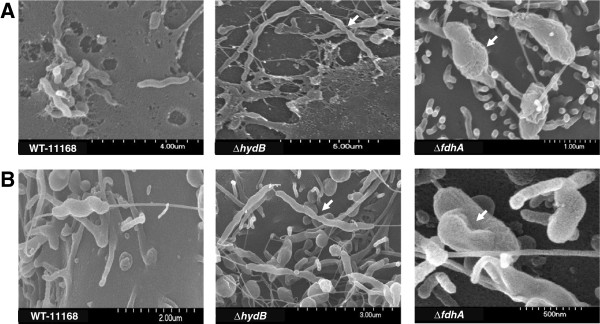
**Scanning electron microscopy analysis of the mutants’ interaction with the PIC and INT-407 cells.** The filamentous and bulging cell shapes (white arrows) associated with the Δ*hydB* and the Δ*fdhA*, respectively, in PIC (**a**) and INT-407 (**b**).

We further assessed the interactions of the mutants with the eukaryotic monolayers using scanning electron microscopy as described elsewhere [31]. As reported by Eucker and Konkel [32], our results show that the INT-407 cells exhibited a typical increase in surface ruffling (formation of a meshwork of appendages and filaments) after the addition of the bacteria as compared to the control (data not shown). However, there were no discernable differences in surface ruffling associated with the addition of the various mutants as compared to that of the wildtype. Surface ruffling was not readily apparent in our PIC and could not be clearly described. Further, while the bacterial cell shape of Δ*napA*, Δ*nrfA*, and Δ*mfrA* did not appear different from that of the wildtype, both Δ*fdhA* (~ 60-70% of the observed cells) and Δ*hydB* (100% of cells) exhibited non-typical phenotypes as compared to the spiral shape of the wildtype cells. Specifically, Δ*hydB* formed elongated filaments that appeared to be made of multiple cells that failed in separation (Figure 4a and b, Table 1), which suggested that the mutant was defective in late cell division. Notably, a similar phenotype was associated with impaired Tat-dependent amidases of *E*. *coli*[33], which are essential for hydrolysis of septal peptidoglycan [33]. In *C*. *jejuni*, the amidase-encoding gene (*amiA*; *Cj1269c*) lacks a Tat signal peptide and is positioned upstream of the cluster that encodes the hydrogenase complex [15,34]. Since some proteins can translocate via the Tat system using the signal peptides of adjacent Tat substrates (hitchhiking), it is possible that the impairment of Hyd (Δ*hydB*) may have resulted in the failure of amidase to translocate to the periplasm [34]. The latter would cause the elongated phenotype observed for Δ*hydB* cells; however, these conclusions require further experimental confirmation. In contrast, the Δ*fdhA* cells were almost spherical showing a characteristic bulging (Figure 4a and b, Table 1), while the precise mechanisms that lead to Δ*fdhA*’s cell morphology are still not clear. Regardless, since the spiral shape of *C*. *jejuni* is important for host colonization [35], we suggest that the morphology of Δ*hydB* and Δ*fdhA* may contribute at least partially to their deficient interactions with PIC and INT-407, respectively. Further, since it is hypothesized that the spiral shape of *C*. *jejuni* may also be associated with its motility in viscous milieus [16], the bulging shape of the Δ*fdhA* might also contribute to its decreased motility (Figure 1a). In addition, it should be noted that follow-up investigations showed that the morphology of Δ*hydB* and Δ*fdhA* was independent of their interactions with the monolayers, because the impaired shapes of the mutants were also observed during growth in Muller-Hinton (MH) broth (data not shown).

Our analysis showed that under all tested conditions (microaerobic vs. anaerobic and 37°C vs. 42°C), Δ*napA*, Δ*nrfA*, Δ*mfrA*, and Δ*fdhA* were not deficient in growth as compared to the wildtype (data not shown). However, the Δ*hydB* exhibited a slight but significant decrease in growth only under anaerobic conditions after 24 h of incubation (data not shown). Therefore, the phenotypes reported for the RP mutants in this study were not affected by the growth properties of the cognate strains. Further, previous studies, gene organization analysis, and our complementation studies showed that the phenotypes reported in this study were not impacted by Polar effects. Specifically, qRT-PCR analysis showed that the transcript levels of *Cj0786* and *Cj0787*, genes that encode a hydrophobic protein and a hypothetical protein, respectively, and are located down-stream of the *nap* operon (*napAGHBLD*) were not affected by the cognate mutation [8]. A similar observation was noted for *Cj1356c*, which encodes an integral membrane protein and is located downstream of *nrfA*[8]. Further, the gene directly downstream of the *mfr* operon, *Cj0440c* (encodes a TenA/Thi-4 family protein) [9], and *Cj1263* (encodes recombination protein RecR) which is downstream of the *hyd* operon are likely not affected by the deletion of *mfrA* and *hydB*, respectively, as they are divergently transcribed [9]. The *fdh* genes are divided into two operons that are transcribed in the same orientation and separated by ~ 67 nucleotides. The operon downstream of *fdhA* contains *fdhD* and *Cj1507c* (encodes the DNA binding protein ModE) [36]. However, the introduction of the individual native genes into the Δ*fdhA* as well as the other RPs mutants resulted in the complementation of the impacted phenotypes (motility, H_2_O_2_ resistance and biofilm formation) (Additional file 1: Table S1).

## Conclusions

In this study, we showed that RPs contribute differentially to key *C*. *jejuni* phenotypes in a manner that depends on the temperature and/or oxygen content of the environment (Table 1). Consequently, we conclude that these proteins partially bestow *C*. *jejuni* with its remarkable ability to adapt and survive in a variety of niches, a characteristic that is crucial for understanding this bacterium’s prevalence, persistence and success as a pathogen.

## Methods

### Bacterial strains and growth conditions

RPs mutants were previously generated in the *C*. *jejuni* NCTC-11168 background and included Δ*napA* (encoding a subunit of the nitrate reductase), Δ*nrfA* (encoding a subunit of the nitrite reductase), Δ*fdhA* (encoding a subunit of the formate dehydrogenase), Δ*hydB* (encoding a subunit of the hydrogenase), and Δ*mfrA* (encoding a subunit of the methylmenaquinol:fumarate reductase) [8-10]. All strains were cultured on MH agar under microaerobic conditions (85% N_2_, 10% CO_2_, 5% O_2_). Incubation at 37°C or 42°C was performed for comparison between temperatures, while oxygen-limited conditions were generated using the BD GasPak Sachets system, which constitutes an atmosphere of less than 1% oxygen and greater than or equal to 13% carbon dioxide (BD diagnostics, NJ, USA). In this paper, oxygen-limited atmosphere was designated as anaerobic to make a clear distinction with microaerobic conditions. Leaked horse blood (5%, Oxoid, KS, USA), antibiotics (chloramphenicol: 20 μg.ml^-1^), and the *Campylobacter* selective supplement (SR155E, Oxoid, KS, USA) were added to the MH medium when necessary. For growth curve analysis, the mutants and wildtype strain were inoculated into MH broth and incubated shaking (200 rpm) at different temperature and oxygen conditions. Growth was monitored by measuring optical density (λ = 600 nm) at different time points.

### Construction of complementation strains

To construct complementation strains, individual native RPs genes (*napA*, *nrfA*, *mfrA*, *hydB*, and *fdhA*) along with their potential promoter sequences were amplified from the genomic DNA of *C*. *jejuni* NCTC-11168 using specific primers (Additional file 2: Table S2). The primers were designed to include restriction sites that facilitate directional cloning. The PCR products were digested, purified and ligated into a similarly digested pRY108 plasmid using a Fast-Link DNA ligation kit (Epicentre). The ligated product was then cloned into Library Efficiency DH5α *E*. *coli* competent cells (Invitrogen, WA, USA). The resulting plasmids were then purified and introduced into the cognate mutant strains by electroporation as described previously [37]. Electroporated cells were spread on MH agar plate supplemented with kanamycin and chloramphenicol and incubated at 42°C for 2 to 3 days under microaerobic conditions. Single colonies representing the complementation strains were streak purified and used for further studies.

### Motility assay

The motility of the RP mutants was determined as described by Fields and Thompson [17]. Briefly, the *Campylobacter* cultures were adjusted to OD_600_ (optical density at λ = 600 nm) of 0.02. Two μl of each culture were then stabbed into semisolid MH plates containing 0.4% agar. The plates were incubated either at 37°C or 42°C under microaerobic conditions. Diameters of the zones of motility were measured after 48 h of incubation. The experiment was repeated at least three times and samples were tested in triplicate. Motility under anaerobic conditions could not be assessed, because the zones of motility were not defined and sufficiently large for reliable measurement.

### Resistance to hydrogen peroxide

The resistance of the RP mutants to H_2_O_2_ (oxidative stress) was determined using a diffusion assay [38]. One-hundred μl of each of the *Campylobacter* cultures (OD_600_ of 1.0) were spread onto MH agar plates. A hole (5 mm in diameter) was aseptically created at the center of the plates and filled with 30 μl of 3% H_2_O_2_[15]. The plates were then incubated at 37°C or 42°C under microaerobic or anaerobic conditions. The diameter of the zone of inhibited growth was measured after 48 h of incubation. All experiments were repeated at least three times and samples were tested in triplicate.

### Biofilm formation assay

The impact of RP deletions on *C*. *jejuni*’s ability to form biofilms was determined using the crystal violet staining assay as described previously [15,17]. Briefly, the *Campylobacter* cultures were suspended in MH broth to achieve an OD_600_ of 0.05. One ml of each culture was transferred to sterile borosilicate glass tubes, which were incubated for 72 h at different conditions. The tubes were then gently washed with distilled water and stained with 0.1% crystal violet for 15 min. After further washing to remove excess stain, the tubes were left to dry at room temperature. The biofilms were then dissolved in 80% DMSO and quantified spectrophotometrically (λ = 570 nm). All experiments were repeated at least three times and samples were tested in triplicate.

### Infection of INT-407 cells

The impact of RP deletions on *C*. *jejuni*’s virulence associated traits was assessed in vitro using human intestinal cells [39,40]. For this purpose, 10^5^ cells ml^-1^ of INT-407 (human embryonic intestine cells, ATCC CCL 6) were seeded into each well of a 24-well tissue culture plates in Minimum Essential Medium Eagle (MEM, Fisher scientific, PA, USA) supplemented with 10% fetal bovine serum (FBS, Fisher scientific, PA, USA). The plates were then incubated at 37°C in a humidified incubator with 5% CO_2_ to obtain semi-confluent mono-layers. Before infection with the *C*. *jejuni* strains, the INT-407 mono-layers were washed three times and covered in MEM supplemented with 1% FBS. Similarly, the *C*. *jejuni* cultures were washed 3 times and suspended in MEM supplemented with 1% FBS to obtain 10^7^ bacteria ml^-1^. One ml of bacterial suspension was added to each well containing the INT-407 semi-confluent monolayer, achieving a 1:100 multiplicity of infection (MOI). To assay for *Campylobacter* adherence, the infected monolayers were incubated for 3 h, which was followed by washing the cells 3 times with 1X PBS, lysis using 0.1% (v/v) Triton X-100 and serial dilution (10-fold) in 1X PBS. One hundred μl of each dilution were spread on MH agar plates. The agar plates were then incubated for 48 h at 42°C under microaerobic conditions after which CFU were counted.

To assay for invasion, infected monolayers were incubated for 3 h, washed 3 times with MEM supplemented with 1% FBS and then treated with gentamicin (150 μg.ml^-1^) for 2 h to inhibit the bacteria that did not invade the cells. At the end of the incubation, the infected mono-layers were washed, lysed, and serial dilutions were plated as described earlier. The number of bacteria that invaded the cells was determined by counting CFUs.

For the intracellular survival assays [41], *Campylobacter* cultures and the INT-407 cells were processed as described above. The monolayers were then covered with MEM containing 1% FBS and gentamicin (10 μg.ml^-1^) and incubated for additional 24 h at 37°C. Following incubation, the monolayers were washed, lysed and processed as described above. The number of viable intracellular bacteria was determined by counting CFUs. For each assay, strains were tested in duplicate, while the experiment was repeated three times on separate occasions.

### Infection of primary chicken intestinal epithelial cells (PIC)

The potential of the RP mutants to adhere to and invade chicken epithelial cells was assessed using primary chicken intestinal epithelial cells (PIC). PICs were isolated using a method described previously [42] with modifications. Briefly, the intestines from 11-day-old chicken embryos (Charles River Laboratories, CT, USA) were harvested and suspended in DMEM supplemented with penicillin and streptomycin (100 U.ml^-1^ and 100 μg.ml^-1^, respectively). Intestines were fragmented into smaller pieces and washed twice with DMEM. Then, the intestinal fragments were placed in a 70 μm nylon mesh filter and gently crushed with a 2 ml syringe piston to obtain a single cell suspension. The cells were then washed twice and the pellet was resuspended in DMEM supplemented with 10% fetal bovine serum and transferred to 25 cm^2^ cell culture flasks. After 7–10 days of incubation, examination using a microscope showed typical cuboidal morphology characteristic of epithelial cells. Before using these cells in the experiments, their purity was confirmed by immunofluoresent examination of the expression of pan-cytokeratin using mouse anti-human pan-cytokeratin mAb (Sigma). Expression of pan-cytokeratin was detected on 100% of the cells assayed (data not shown). PICs were then seeded into 24-well tissue culture plates and assays for adhesion, invasion and intracellular survival of *C*. *jejuni* were performed as described for the INT-407 infection studies.

### Scanning electron microscopy

To further investigate the interaction between the RPs mutants and the INT-407 cells and PIC, infected monolayers were analyzed using scanning electron microscopy (SEM) as described previously [31] with minor modifications. Briefly, different cell types were grown on HCl treated glass coverslips. The *C*. *jejuni* strains were added to the monolayers at an MOI of 200. After 3 h of incubation, the cells were gently washed with 1X PBS and fixed (3% glutaraldehyde, 2% paraformaldehyde in 0.1 M potassium phosphate buffer, pH 7.2) at 4°C overnight. The samples were then rinsed in 0.1 M potassium phosphate (3 times with 15 min incubation for each step) and post-fixed with 1% osmium tetroxide for 1 h at room temperature in the dark. This was followed with serial dehydration of the samples in ethanol, critical point drying and platinum sputter-coating (Molecular and Cellular Imaging Center, Ohio Agricultural Research and Development Center [OARDC]; http://www.oardc.ohio-state.edu/mcic). The samples were visualized and imaged using the Hitachi S-4700 scanning electron microscope. All samples were tested in duplicate and non-infected monolayers were used as controls to assess morphological changes associated with the bacterial infection.

### Statistics

Data were expressed as mean ± SE (standard error) and statistical analysis was performed using the student’s *t*-test. A *P* value of <0.05 was considered statistically significant. Unless otherwise indicated in the text, the reported statistics highlight comparisons between each mutant strain and the wildtype.

## Competing interests

The authors declare that they have no competing interests.

## Authors' contributions

IIK and GR conceived and designed the study. IIK, MK, MAE, and YMS performed the experiments. JWO provided the mutants. IIK and GR wrote the paper. IIK, GR, JWO, MAE and YMS reviewed and edited the manuscript. All authors read and approved the final manuscript.

## Supplementary Material

Additional file 1 **Table S1.** Analysis using the complementation strains shows that the phenotypes were rescued to levels that were comparable to those associated with the wildtype. Not applicable (NA) indicates the instances where the mutant did not show a divergent phenotype, hence the complementation strain was not tested. Data were reported as means and * indicates statistical significance (*P* < 0.05). The complementation of the *fdhA* reverted the deficiency in biofilm formation associated with the Δ*fdhA* to levels that were higher than those of the wildtype. Click here for file

Additional file 2 **Table S2.** List of primers used to generate the complementation strains. Restriction sites are underlined. Click here for file
