# Supplementary material for: Respiratory proteins contribute differentially to Campylobacter jejuni’s survival and in vitro interaction with hosts’ intestinal cells
Source: BMC Microbiol. 2012 Nov 13;12:258. doi: 10.1186/1471-2180-12-258 (PMC3541246; doi:10.1186/1471-2180-12-258)
Supplement: Additional file 1 — Table S1. Analysis using the complementation strains shows that the phenotypes were rescued to levels that were comparable to those associated with the wildtype. Not applicable (NA) indicates the instances where the mutant did not show a divergent phenotype, hence the complementation strain was not tested. Data were reported as means and * indicates statistical significance (P < 0.05). The complementation of the fdhA reverted the deficiency in biofilm formation associated with the ΔfdhA to levels that were higher than those of the wildtype. [file 1471-2180-12-258-S1.docx]

Table S1: Analysis using the complementation strains shows that the phenotypes were rescued to levels that were comparable to those associated with the wildtype. Not applicable (NA) indicate the instances where the mutant did not show a divergent phenotype, hence the complementation strain was not tested. Data was reported as means and * indicates statistical significance (*P*<0.05). The complementation of the *fdhA* reverted the deficiency in biofilm formation associated with the ∆*fdhA* to levels that were higher than those of the wildtype .

|  | **Motility**  **(Micro)** | | **Res. H_2_O_2_**  **(Micro)** | | **Res. H_2_O_2_**  **(Ana)** | | **Biofilm**  **(Micro)** | | **Biofilm**  **(Ana)** | | **Biofilm**  **(O_2_)** | |
| --- | --- | --- | --- | --- | --- | --- | --- | --- | --- | --- | --- | --- |
|  | **Diameter of zone of motility (cm)** | | **Diameter of zone of inhibition (cm)** | | | | **Optical density (λ = 570 nm)** | | | | | |
|  | **37° C** | **42° C** | **37° C** | **42° C** | **37° C** | **42° C** | **37° C** | **42° C** | **37° C** | **42° C** | **37° C** | **42° C** |
| ***C. jejuni* NCTC-11168** | 4.9 | 6.7 | 5.0 | 4.85 | 5.15 | 4.9 | 2.27 | 2.3 | 1.7 | 1.14 | 2.18 | 1.7 |
| **Complementation strains** |  | |  | | | |  | | | | | |
| ***C-∆napA*** | 5.6 | 6.6 | 5.0 | 4.8 | 5.6 | 4.9 | NA | NA | 1.79 | NA | 1.98 | 1.6 |
| ***C-∆nrfA*** | 4.9 | 6.6 | NA | NA | NA | NA | NA | NA | 1.78 | NA | NA | NA |
| ***C-∆mfrA*** | 5.2 | 7.0 | 5.0 | 5.2 | 5.15 | 4.4 | 2.2 | 2.2 | NA | 1.1 | NA | NA |
| ***C-∆hydB*** | NA | NA | NA | NA | NA | NA | NA | NA | NA | NA | NA | NA |
| ***C-∆fdhA*** | 4.7 | 6.1 | 5.6 | 5.1 | 5.1 | 5.1 | 2.6* | NA | NA | NA | NA | NA |
