# Supplementary material for: Respiratory proteins contribute differentially to Campylobacter jejuni’s survival and in vitro interaction with hosts’ intestinal cells
Source: BMC Microbiol. 2012 Nov 13;12:258. doi: 10.1186/1471-2180-12-258 (PMC3541246; doi:10.1186/1471-2180-12-258)
Supplement: Additional file 2 — Table S2. List of primers used to generate the complementation strains. Restriction sites are underlined. [file 1471-2180-12-258-S2.doc]

Table S2: List of primers used to generate the complementation strains. Restriction sites are underlined.

| **Primer name** | **Primer sequence** | **Restriction enzyme** |
| --- | --- | --- |
| C-*nap*-F  C-*nap*-R | 5’-TATCGGATCCAATTTTGATTAAGCCCATTC-3’  5’-TCGTGGTACCTTAATCAAAACCCTAAATTA-3’ | *BamH*I  *Kpn*I |
| C-*nrf*-F  C-*nrf*-R | 5’-ACGTGGTACCTGTGATATTTTGACAAAACA-3’  5’-CTGAGGATCCATAAACATATCAGGACTTGT-3’ | *Kpn*I  *BamH*I |
| C-*mfr*-F  C-*mfr*-R | 5’-ACGAGGATCCAATGCAATTTATGAATGGAG-3’  5’-GTCAGGTACCACAAATTGCAGATTGACAAG-3’ | *BamH*I  *Kpn*I |
| C-*fdh*-F  C-*fdh*-R | 5’-CTCAGGTACCCACAACCTATGCAAATTTCT-3’  5’-TCCAGGATCCCTTTGATTTCTAGGGTAGCA-3’ | *Kpn*I  *BamH*I |
| C-*hyd*-F  C-*hyd*-R | 5’-TGCAGGTACCAGTACAAACTCCGCAAATTC-3’  5’-CGTCGGATCCTATTCATCCTGCAAGATTTT-3’ | *Kpn*I  *BamH*I |
